# Analysis of mitral valve regurgitation by computational fluid dynamics

**DOI:** 10.1063/1.5097245

**Published:** 2019-08-23

**Authors:** Dario Collia, Luigino Zovatto, Gianni Pedrizzetti

**Affiliations:** Department of Engineering and Architecture, University of Trieste, P. Europa 1, 34127 Trieste, Italy

## Abstract

The clinical syndrome of mitral insufficiency is a common consequence of mitral valve (MV) prolapse, when the MV leaflets do not seal the closed orifice and blood regurgitates back to the atrium during ventricular contraction. There are different types of MV prolapse that may influence the degree of regurgitation also in relation to the left ventricle (LV) geometry. This study aims to provide some insight into the fluid dynamics of MV insufficiency in view of improving the different measurements available in the clinical setting. The analysis is performed by a computational fluid dynamics model coupled with an asymptotic model of the MV motion. The computational fluid dynamics solution uses the immersed boundary method that is efficiently integrated with clinical imaging technologies. Healthy and dilated LVs obtained by multislice cardiac MRI are combined with simplified models of healthy and pathological MVs deduced from computed tomography and 4D-transesophageal echocardiography. The results demonstrated the properties of false regurgitation, blood that did not cross the open MV orifice and returns into the atrium during the backward motion of the MV leaflets, whose entity should be accounted when evaluating small regurgitation. The regurgitating volume is found to be proportional to the effective orifice area, with the limited dependence of the LV geometry and type of prolapse. These affect the percentage of old blood returning to the atrium which may be associated with thrombogenic risk.

## I. INTRODUCTION

Mitral valve prolapse (MVP) is the common cause of the mitral regurgitation (MR) that affects approximately 2.4% of the population,^10^ which is typically caused by myxomatous degeneration of the valve.^20^ Clinical classification of MR is based Carpentier's^1^ functional characterization of the leaflets, which identifies the anterolateral, medial, and posteromedial cusps of the posterior leaflet as P1, P2, and P3 and the anterior leaflet as A1, A2, and A3. It classifies MR of the individual leaflet based on its movements in three types. In type I MR, the leaflets have a normal movement and regurgitation is imputable to annular dilatation and incomplete coaptation of the leaflet or insufficient surface of coaptation; in type II MR, the leaflets are prolapsed or hypermobile, which can be caused by elongation or rupture of chordae tendineae or by lengthening or rupture of the papillary muscles. Finally, in type III MR, the leaflets have a limited movement caused by their stiffening and retraction or calcification.

Different methods have been introduced for evaluating MR. The vena contracta method^19^ estimates the size of the regurgitant orifice by measuring the minimum diameter of the regurgitant blood jet; then, small measurement errors correspond to larger volumetric quantification errors, which represent a limitation given by a large inter- and intraoperator variability.^24^ The Proximal Isosurface Velocity Area (PISA) method evaluates the MR volume based on the 2D Color-Doppler image of the converging flow proximal to the orifice, the velocity is shown with different colors, and the assumption of hemispheric converging flow allows estimating the flow rate based on the value of one isovelocity contour. This method is the most utilized for its simplicity, but measures are often subject to error^18^ because the regurgitant area is not hemispherical; often, it is at half-moon or elliptical; moreover, the PISA calculation relies on instantaneous measures that are extended to the cardiac cycle for a volumetric estimation (typically assuming a proportion with the velocity trace measured with Doppler at the LV outflow). In recent years, the increasing diffusion of cardiac magnetic resonance (CMR) allows more accurate evaluations of MR. High quality phase-contrast CMR with velocity-encoded imaging is used for estimating the aortic flow and calculates the absolute regurgitation volume from the difference volume rate in the left ventricle (LV). This approach is considered the most reliable although it calculates MR from a difference, rather than directly on the mitral valve (MV), presenting some degree of variability.^17^ The effective orifice area (EOA)^8,13,14^ represents a more structural measure of MV insufficiency; this approach also presents various limitations^3^ partly imputable to the difficulty of measuring the EOA directly from images of the MV orifice (MVO).

Although these methods provide an assessment for MR in the routine clinical environment, the description of the flow phenomena involved in MR is certainly incomplete and several dysfunctional states can largely differ in the fluid dynamics about the MV despite similar levels of MR or similar symptoms with different MR patterns.

This study aims to get deeper insight into the flow phenomena associated with MR. This is achieved by reproducing, using direct numerical simulation (DNS), the fluid dynamics in the LV in correspondence with pathologic MVPs obtained from clinical images. The focus of this study is on blood motion, and a simplified model for flow-driven MV motion is employed. This approach allows estimating the cardiac fluid dynamics associated with different types and gravity of MVPs and how the actual regurgitated volume is correlated with EOA. It will analyze the wash-out of the LV as well as the residence time properties of the regurgitant blood that could reflect in additional risk factors.^11,12,22^

## II. RESULTS

### A. Fluid dynamics in healthy and dilated LV

In this section, we describe the fluid dynamics that develops in normal and dilated LV geometries in the presence of the different MV morphologies described in Sec. IV A. The normal LV is characterized by EDV=113.47 ml, ESV=46.52 ml, Stroke volume (SV)=66.95 ml, and ejection-fraction $\mathrm{EF}=\frac{\mathrm{SV}}{\mathrm{EDV}}=59\%$.

**FIG. 1.**
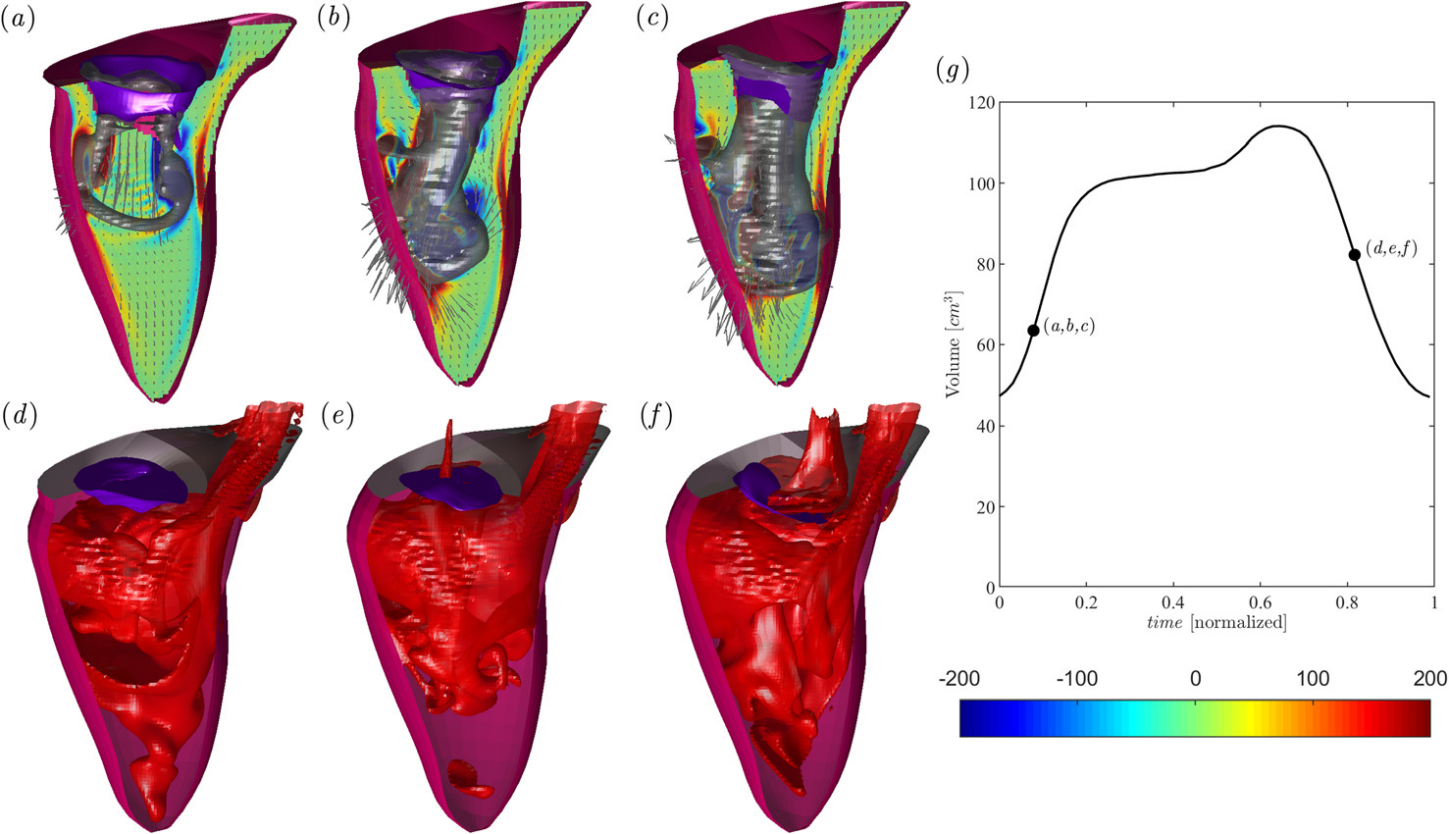
Flow field in the normal LV in the presence of a healthy MV [(a), and (d)] and MV with P3 Prolapse [(b), and (e)] and P2 Prolapse [(c), and (f)]. Diastolic flow at the peak E-wave (a), (b), and (c), as indicated in the volume curve inset [black line in (g)]; the normal vorticity is shown in red to blue color from –200 units to 200 units equal to the inverse of the heartbeat period and the velocity vector (every 4 grid points) on a longitudinal plane crossing the center of MV of aorta and the LV apex; the three-dimensional gray surfaces represent one isosurface of the $\lambda_{2}$ parameter. The lower side is the three-dimensional flow field in the healthy LV calculated using the passive scalar isosurface method at the same instants of early systole at a value of C = 0.4, as indicated in the volume curve inset. The inset in (g) reports the volume curve and the instant of the case analyzed.

The flow field in the normal LV at peak diastole is shown in Figs. 1(a)–1(c) in correspondence with (a) Healthy, (b) P3, and (c) P2 MVs. The flow with healthy MV presents the typical asymmetric vortex ring that enters the LV, the P3 case shows a vortex ring that deviates and breaks down when impacting the posterior wall, and finally, the mitral jet in the P2 MV is characterized by an irregular flow that enters in depth into the center of the LV probably because of the slightly reduced dimension of the MVO. Figures 1(d)–1(f) reports the corresponding concentration field at peak systole. The healthy case evidences blood that is well mixed between old and fresh blood in the entire chamber; differently, the prolapse MVs are associated with the greater presence of old blood near the apex and displays regurgitation in atrium.

**FIG. 2.**
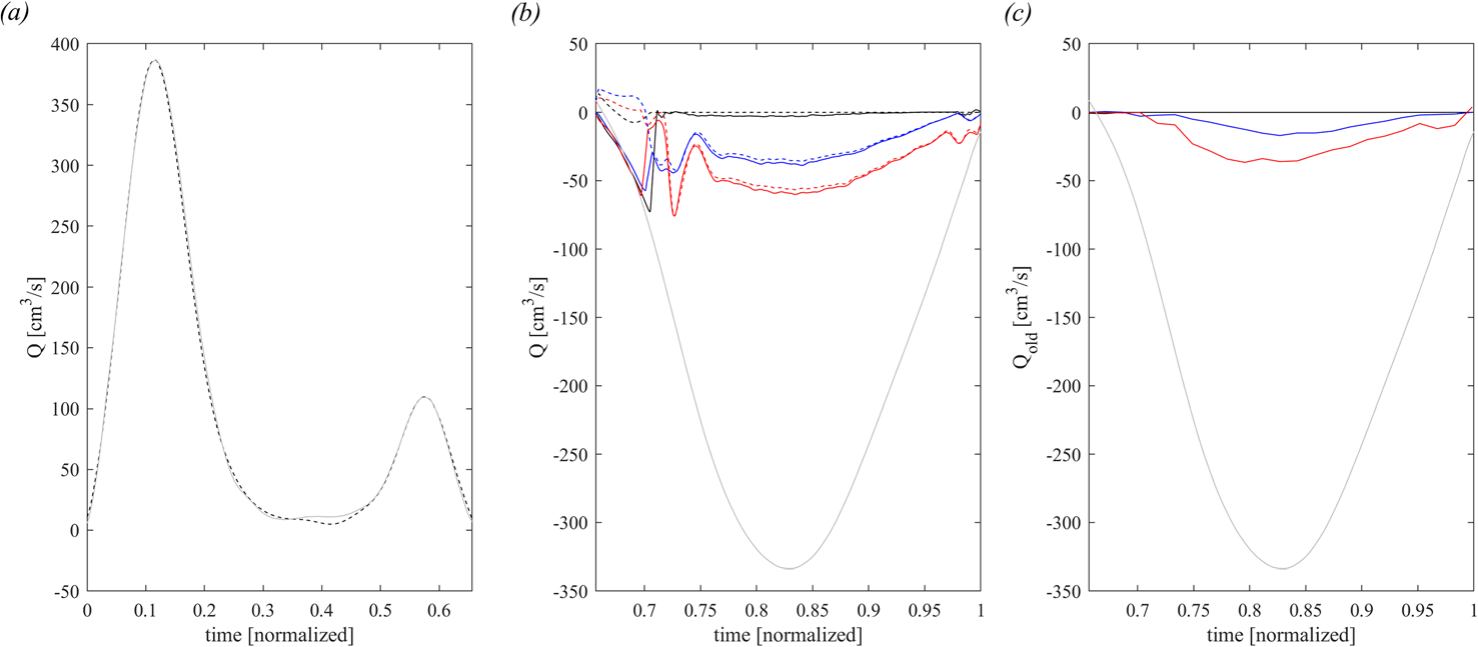
Flow balance in diastole. (a) Comparison between diastolic dV/dt (dashed dark line) and diastolic $Q_{\mathrm{LV}}$ (continuous gray line) in healthy LV. In systole, (b) comparison of $Q_{\mathrm{MVa}}$ (continuous line) and $Q_{\mathrm{MV}}$ (dashed line) for healthy-1 MV (dark line), P3 MVP (blue line), and P2 MVP (red line) and the gray continuous line is the systolic dV/dt, (c) old blood flow regurgitated in atrium, computed by $Q_{{\mathrm{MV}}_{\mathrm{old}}}$ for healthy MV (dark line), P3 MVP (blue line), and P2 MVP (red line); the gray continuous line is the systolic dV/dt.

The flow profile, dV/dt, for the healthy LV is shown in Fig. 2(a) during diastole, and in Fig. 2(b) during systole (light gray curves), their integral represents the stroke volume of 66.95 cm^3^. For comparison, Fig. 2(a) also reports the time profile of $Q_{\mathrm{LV}}$ computed using Eq. (12), which gives and integral $V_{\mathrm{LV}}=$ 67.25 cm^3^. Such agreement between the volume change and the measured flow rate represents an indirect agreement of the reliability of the present IBM calculation.

The systolic outflow in aorta in Fig. 2(b) is plotted together the blood flow that crosses the LV annulus and returns to the atrium, $Q_{\mathrm{MVa}}$ computed by (10) (continuous lines), and with the blood flow that crosses the MV orifice, $Q_{\mathrm{MV}}$ computed by (8) (dashed lines), for normal (healthy: black color) and prolapsed MVs (P3: blue, and P2: red), respectively. The first part of systole corresponds to the closing period of the MV, and here, the continuous and dashed curves differ because $Q_{\mathrm{MVa}}$ includes the volume of blood contained in the MV pool above the orifice that returns back to the atrium during MV closure; afterward, the curves come together along a common path. The initial difference represents the so-called false regurgitation that identifies the physiological blood flow returning in the atrium during the closure of the MV leaflets, which is estimated by the difference $V_{{\mathrm{reg}}_{\mathrm{false}}}=V_{{\mathrm{reg}}_{\mathrm{MVa}}}-V_{\mathrm{reg}}$ computed using (11) and (9). The same curves of regurgitating flow are shown in Fig. 2(c) limiting the calculation to the old blood only, computed using Eq. (14). The overall entity or old blood regurgitation is nearly proportional to the total regurgitations; nevertheless, it is evident that the old blood enters the atrium mainly during the second half of systole when ejection has involved the entire LV volume. The initial regurgitation is mainly composed of fresh blood that reached the region just behind the valve and is the first involved in the back flow.

The integral results relative to all the different MVs are reported in Table I. The healthy valve is a physiologic MV that presents a nonzero EOA = 0.06 cm^2^, corresponding to a tiny hole with a 1% valve size that is not caused by pathological valvular deformation or imputable to minor inaccuracy in image segmentation. The results show how the increase in the EOA corresponds to an increase in $V_{\mathrm{reg}}$, whose correlation will be analyzed later. Regurgitation reaches about 10% of the SV in the present P3 prolapse and about 20% in P2. It is noticed that a small nonzero regurgitation is also found in healthy valves, indicating that MV velocity during closure is slightly lower than blood velocity. The false regurgitation, $V_{{\mathrm{reg}}_{\mathrm{false}}}{=V}_{{\mathrm{reg}}_{\mathrm{MVa}}}-V_{\mathrm{reg}}$, represents the fresh atrial blood that does not cross the MV orifice and returns into the atrium at the beginning of systole. This does not depend on the EOA and is more a consequence of the MV shape.

**TABLE I.** Global results in healthy LV. MVA, mitral valve area; EOA, effective orifice area at the MV closed position; $\frac{\mathrm{EOA}}{\mathrm{MVA}}$, EOA normalized with MVA; SV, Stroke volume; $V_{{\mathrm{reg}}_{\mathrm{MVa}}}$, total regurgitation; $V_{{\mathrm{reg}}_{\mathrm{false}}}$, false regurgitation; $V_{\mathrm{reg}}$, regurgitation from orifice; and $\frac{V_{\mathrm{reg}}}{\mathrm{SV}}$, $V_{\mathrm{reg}}$ normalized with SV.

	$\mathrm{MVA}\;({\mathrm{cm}}^{3})$	$\mathrm{EOA}\;({\mathrm{cm}}^{3})$	$\frac{\mathrm{EOA}}{\mathrm{MVA}}\;(\%)$	$\mathrm{SV}({\mathrm{cm}}^{3})$	$V_{{\mathrm{reg}}_{\mathrm{MVa}}}\;({\mathrm{cm}}^{3})$	$V_{{\mathrm{reg}}_{\mathrm{false}}}\;({\mathrm{cm}}^{3})$	$V_{\mathrm{reg}}\;({\mathrm{cm}}^{3})$	$\frac{V_{\mathrm{reg}}}{\mathrm{SV}}$ (%)
Healthy	**6.20**	**0.06**	1	66.95	2.07	1.64	0.43	0.65
P3	**4.83**	**0.52**	11	66.95	8.87	2.58	6.29	9.40
P2	**4.26**	**0.87**	20	66.95	13.42	0.66	12.76	19.07
P3-25%	**4.83**	**0.39**	8	66.95	4.57	2.49	2.08	3.11
P3-50%	**4.83**	**0.26**	5	66.95	2.58	1.00	1.58	2.37
P3-Healthy	**4.83**	**0**	0	66.95	1.70	0.90	0.80	1.20

Table II shows the volume of residual old blood stagnant into the LV at end systole and the volume of old blood regurgitated in atrium. These results show that the residual volume $V_{\mathrm{residual}}$ is only moderately influenced by the MV regurgitation that, therefore, does not directly influence the LV wash-out. On the other hand, the increase in regurgitation is associated with a larger percentage of old blood regurgitated that reaches a value as high as 50% in the most diseased case.

**TABLE II.** Global transit results in healthy LV. EOAis the effective orifice area at the MV closed position, $V_{\mathrm{residual}}$ is the LV residual volume at end-systole, $V_{{\mathrm{reg}}_{\mathrm{old}}}$ is the quantity of the old blood regurgitated in atrium, $\frac{V_{{\mathrm{reg}}_{\mathrm{old}}}}{V_{\mathrm{reg}}}$ is the old blood regurgitated normalized with $V_{\mathrm{reg}}$, and $V_{\mathrm{residual}}$ is the residual volume.

	$\mathrm{EOA}\;({\mathrm{cm}}^{2})$	$V_{\mathrm{residual}}$ (%)	$V_{{\mathrm{reg}}_{\mathrm{old}}}$ $\left({\mathrm{cm}}^{2}\right)$	$\frac{V_{{\mathrm{reg}}_{\mathrm{old}}}}{V_{\mathrm{reg}}}$ (%)
Healthy	0.06	28.50	0.03	6.98
P3	0.52	26.07	2.36	37.52
P2	0.87	30.67	6.42	50.31
P3-25%	0.39	25.28	0.70	33.65
P3-50%	0.26	25.60	0.09	5.70
P3-Healthy	0	26.12	0.01	1.25

The same analysis is repeated in correspondence with a pathological ventricular geometry corresponding to a severe dilated cardiomyopathy. The dilated LV is characterized by an enlarged end-diastolic volume EDV=199.31 ml, end-systolic volume (ESV)=142.50 ml, SV=56.82 ml, and ejection-fraction EF=29%.

**FIG. 3.**
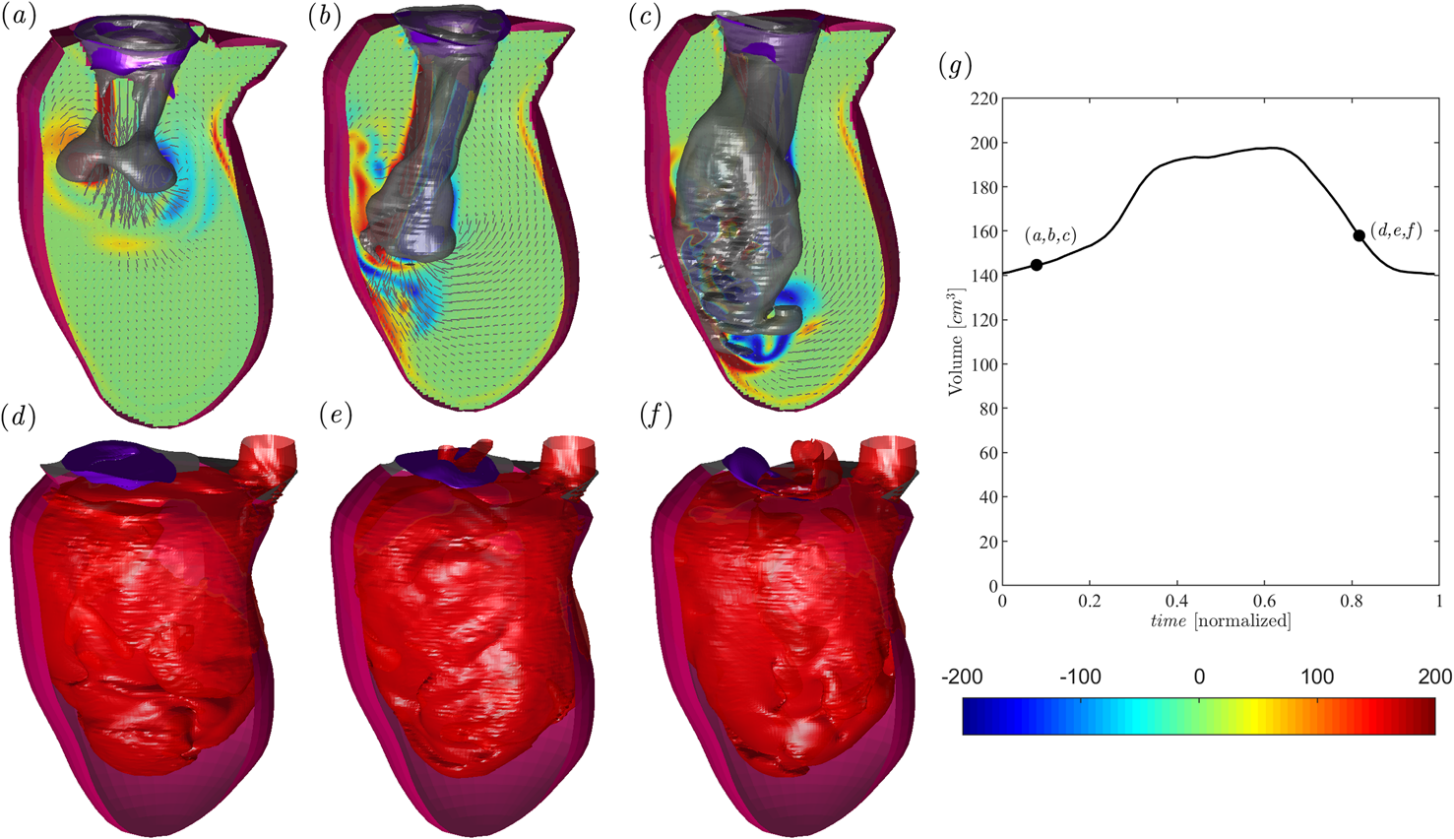
Flow field in the dilated LV in the presence of a healthy MV [(a) and (d)] and MV with P3 prolapse [(b) and (e)] and P2 prolapse [(c) and (f)]. Diastolic flow at the peak E-wave (a)–(c), as indicated in the volume curve inset [black line in (g)]; the normal vorticity is shown by red to blue color from –200 units to 200 units equal to the inverse of the heartbeat period and the velocity vector (every 4 grid points) on a longitudinal plane crossing the center of MV of aorta and the LV apex; the three-dimensional gray surfaces represent one isosurface of the $\lambda_{2}$ parameter. The lower side is the three-dimensional flow field in the dilated LV calculated using the passive scalar isosurface method at the same instants of early systole at a value of C = 0.4, as indicated in the volume curve inset. The inset (g) reports the volume curve and the instant of the case analyzed.

The flow field in the dilated LV at peak diastole is shown in Figs. 3(a)–3(c) in correspondence with (a) healthy, (b) P3, and (c) P2 MVs. The overall intraventricular fluid dynamics in dilated ventricles was previously described in the literature,^16,23^ and it is qualitatively confirmed by the present results. All dynamic phenomena are weaker because velocities are lower, and a slow circulation persists during a large part of the heart cycle. In the dilated LV with healthy MV [Fig. 3(a)], the early diastolic jet enters into a chamber with quasiquiescent fluid, and the ring propagates deeper in the large LV before it interacts with the walls and partly dissipates. In the presence of pathological MV [Figs. 3(b) and 3(c)], the mitral jet and the vortex ring at the jet's head present the same deviation previously noticed in the normal LV, which is caused by the arrangement of the MVO. Figures 3(d)–3(f) report the corresponding concentration field at peak systole. Blood is well mixed between old and fresh blood but with the more marked presence of old blood in the entire chamber caused by a larger size of the pathological ventricle and reduced wash-out.

**FIG. 4.**
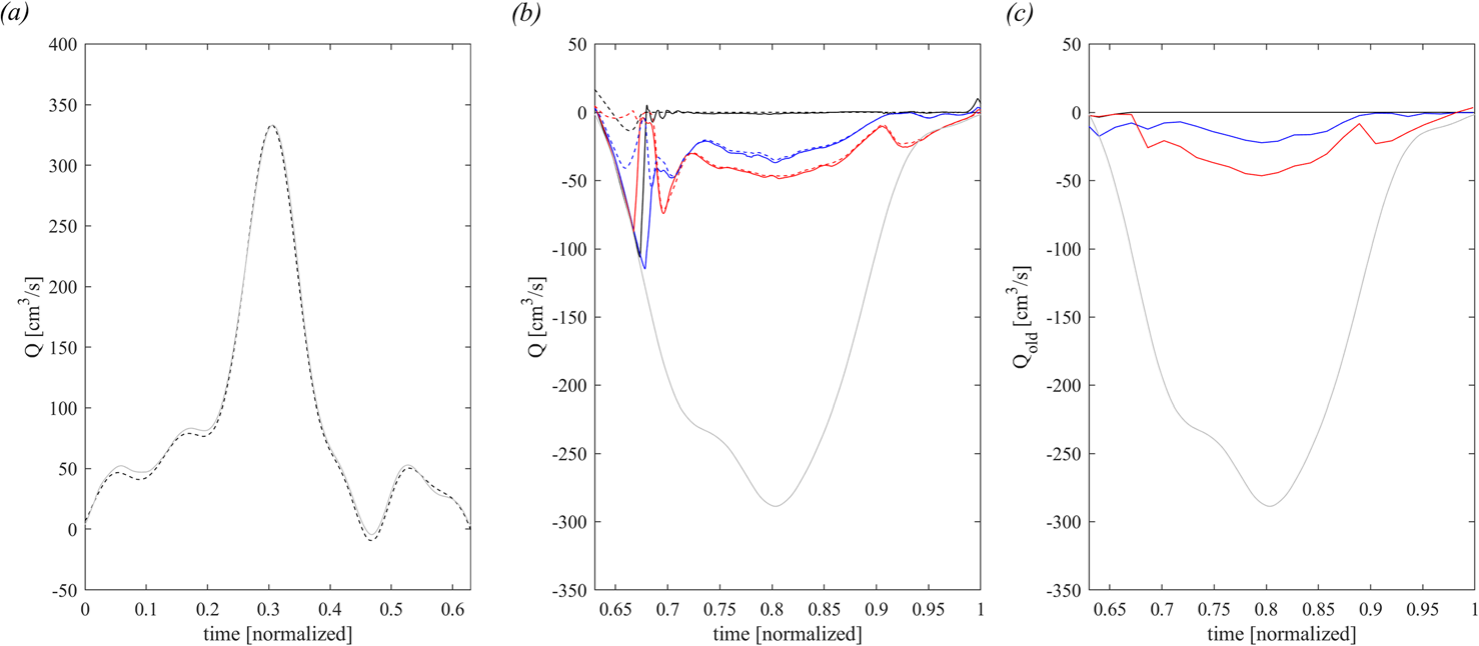
Flow balance in diastole. (a): comparison between diastolic dV/dt (dashed dark line) and diastolic $Q_{\mathrm{LV}}$ (continuous gray line) in dilated LV. In systole, (b): comparison of the $Q_{\mathrm{MVa}}$ (continuous line) and $Q_{\mathrm{MV}}$ (dashed line) for healthy-1 MV (dark line), P3-3 MVP (blue line), and P2 MVP (red line); the gray continuous line is the systolic dV/dt, (c): old blood flow regurgitated in atrium, computed by $Q_{{\mathrm{MV}}_{\mathrm{old}}}$ for healthy-1 MV (dark line), P3-3 MVP (blue line), and P2 MVP (red line); the gray continuous line is the systolic dV/dt.

The flow profile, dV/dt, for the dilated LV is shown in Fig. 4(a) during diastole and in Fig. 4(b) during systole (light gray curves); their integral represents the stroke volume of 56.82 cm^3^. For comparison, Fig. 4(a) reports the time profile of $Q_{\mathrm{LV}}$ computed using Eq. (12), which gives and integral $V_{\mathrm{LV}}=$ 56.82 cm^3^. The systolic outflow in Fig. 4(b) is computed and reported using the same procedure described above for the normal LV and confirms the presence of false regurgitation at the beginning of systole. Similarly, the quantity of old blood regurgitated, shown in Fig. 4(c), presents a trend that is similar to the normal case.

Quantitative evaluations are obtained by the integral results reported in Tables III and IV (to be compared with Tables I and II relative to normal LV). The values of regurgitation are comparable between normal and dilated conditions in correspondence with the saved MV. This is true both in absolute and relative terms, considering that the two LVs have a similar SV. A remarkable difference is found in terms of wash-out, where the dilated LV presents a much larger residual volume, independent of the MV. Also, the quantity of old blood regurgitated is partially increased, indicating that some larger amount of old blood returns back to the atrium and further reduces the overall washout of the entire left heart, thus theoretically increasing the risk of platelet activation and thrombus formation.

**TABLE III.** Global results in dilated LV. MVA, mitral valve area; EOA, effective orifice area at the MV closed position; $\frac{\mathrm{EOA}}{\mathrm{MVA}}$, EOA normalized with MVA; SV, Stroke volume; $V_{{\mathrm{reg}}_{\mathrm{MVa}}}$, total regurgitation; $V_{{\mathrm{reg}}_{\mathrm{false}}},$ false regurgitation; $V_{\mathrm{reg}}$, regurgitation from orifice; and $\frac{V_{\mathrm{reg}}}{\mathrm{SV}}{,\;V}_{\mathrm{reg}}$ normalized with SV.

	$\mathrm{MVA}\;({\mathrm{cm}}^{2})$	$\mathrm{EOA}\;({\mathrm{cm}}^{2})$	$\frac{\mathrm{EOA}}{\mathrm{MVA}}\;\;\left(\%\right)$	$\mathrm{SV}\;\left({\mathrm{cm}}^{3}\right)$	$V_{{\mathrm{reg}}_{\mathrm{MVa}}}\;\;({\mathrm{cm}}^{3})$	$V_{{\mathrm{reg}}_{\mathrm{false}}}\;({\mathrm{cm}}^{3})$	$V_{\mathrm{reg}}\;({\mathrm{cm}}^{3})$	$\frac{V_{\mathrm{reg}}}{\mathrm{SV}}$ (%)
Healthy	**6.20**	**0.06**	1	56.82	2.41	2.40	0.10	0.18
P3	**4.83**	**0.52**	11	56.82	9.27	2.01	7.26	12.77
P2	**4.26**	**0.87**	20	56.82	11.74	0.04	11.70	20.60
P3-25%	**4.83**	**0.39**	8	56.82	5.78	1.96	3.82	6.73
P3-50%	**4.83**	**0.26**	5	56.82	3.53	2.05	1.48	2.61
P3-Healthy	**4.83**	**0**	0	56.82	3.02	2.01	1.01	1.77

**TABLE IV.** Global transit results in dilated LV. EOA is the effective orifice area at the MV closed position, $V_{\mathrm{residual}}$ is the LV residual volume at end-systole, $V_{{\mathrm{reg}}_{\mathrm{old}}}$ is the quantity of the old blood regurgitated in atrium, $\frac{V_{{\mathrm{reg}}_{\mathrm{old}}}}{V_{\mathrm{reg}}}$ is the old blood regurgitated normalized with $V_{\mathrm{reg}}$, and $V_{\mathrm{residual}}$ is the residual volume.

	$\mathrm{EOA}\;({\mathrm{cm}}^{2})$	$V_{\mathrm{residual}}\;(\%)$	$V_{{\mathrm{reg}}_{\mathrm{old}}}$ $\left({\mathrm{cm}}^{2}\right)$	$\frac{V_{{\mathrm{reg}}_{\mathrm{old}}}}{V_{\mathrm{reg}}}$ (%)
Healthy	0.06	68.50	0.01	10
P3	0.52	69.79	3.74	51.51
P2	0.87	70.90	8.33	71.20
P3-25%	0.39	69.70	1.88	49.21
P3-50%	0.26	69.49	0.57	38.51
P3-Healthy	0	69.02	0.61	60.4

**FIG. 5.**
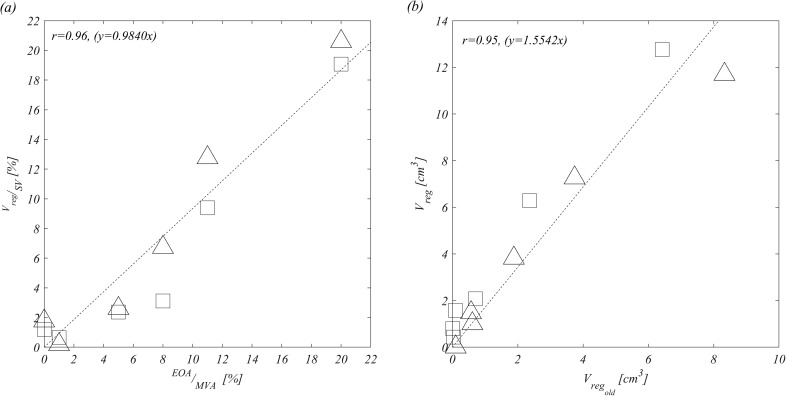
Correlation between $\frac{V_{\mathrm{reg}}}{\mathrm{SV}}$ and $\frac{\mathrm{EOA}}{\mathrm{MVA}}$ considering all MV cases and healthy LV (square) and dilated LV (triangle) (a) and $V_{\mathrm{reg}}$ and $V_{{\mathrm{reg}}_{\mathrm{old}}}$ considering all MV cases and healthy (square) and dilated LV (triangle) (b).

Figure 5(a) reports the correlation between the functional measure of valvular insufficiency, given by the regurgitant volume, and the structural measure, given by the EOA. Once these values are properly normalized, the two quantities present a direct proportionality,

$$\frac{V_{\mathrm{reg}}}{\mathrm{SV}}≅\frac{\mathrm{EOA}}{\mathrm{MVA}},$$ (1)

with good accuracy without evident differences for the different types of prolapse or the LV size. Figure 5(b) shows a good correlation between the total regurgitation and the portion made of old blood; however, some difference is noticeable between normal and dilated LVs as previously underlined.

## III. CONCLUSION

This numerical study provides some insights into the fluid dynamics of mitral valve regurgitations. The calculation employed a simplified model of MV deformation, based on clinical images, which was found to be appropriate for flow modeling purposes. The analysis has been performed for different prolapsed MVs inserted in both a normal and a dilated LV.

First, the study evidenced the presence of a false regurgitation made of blood that was in the MV cup and returned the atrium during MV closure. In small prolapses, this false volume can be comparable to the real regurgitation and should be considered in clinical measurements.

The amount of regurgitation is principally due to the effective insufficiency of the MV without a significant influence of the geometry of the LV. The amount of regurgitating volume, expressed in percentage of the stroke volume, is found to be an equivalent measure of the effective orifice area, expressed in percentage of the MV area. The dimensions of the LV enter into play in terms of the quality of the regurgitant blood; larger LVs present a reduced wash-out that reflects in a larger percentage of old blood returning to the atrium, thus increasing the risk of blood aggregation and thrombus.

## IV. METHODS

### A. Medical imaging and geometries

The healthy and dilated LV geometries used for numerical simulation are obtained by 3D multislice CMR by combining three long-axis borders corresponding to the 2-, 3-, and 4-chamber projections as previously described.^21^ The entire LV endocardial surface is then described by its 3D coordinates evaluated by interpolation on a structured mesh made of 768 points along the circumference and 384 points from the base to the apex; however, the results were independent of the specific number of interpolation points.^21^ The LV geometry during all phases of the heartbeat is then described by the position vector X(ϑ,s,t) of its endocardial surface, where the structured parametric coordinates, (ϑ,s), run along the circumference and from the base to the apex, respectively, and t is the time. The position vector marks LV material points, and their velocity is obtained by time differentiation.

**FIG. 6.**
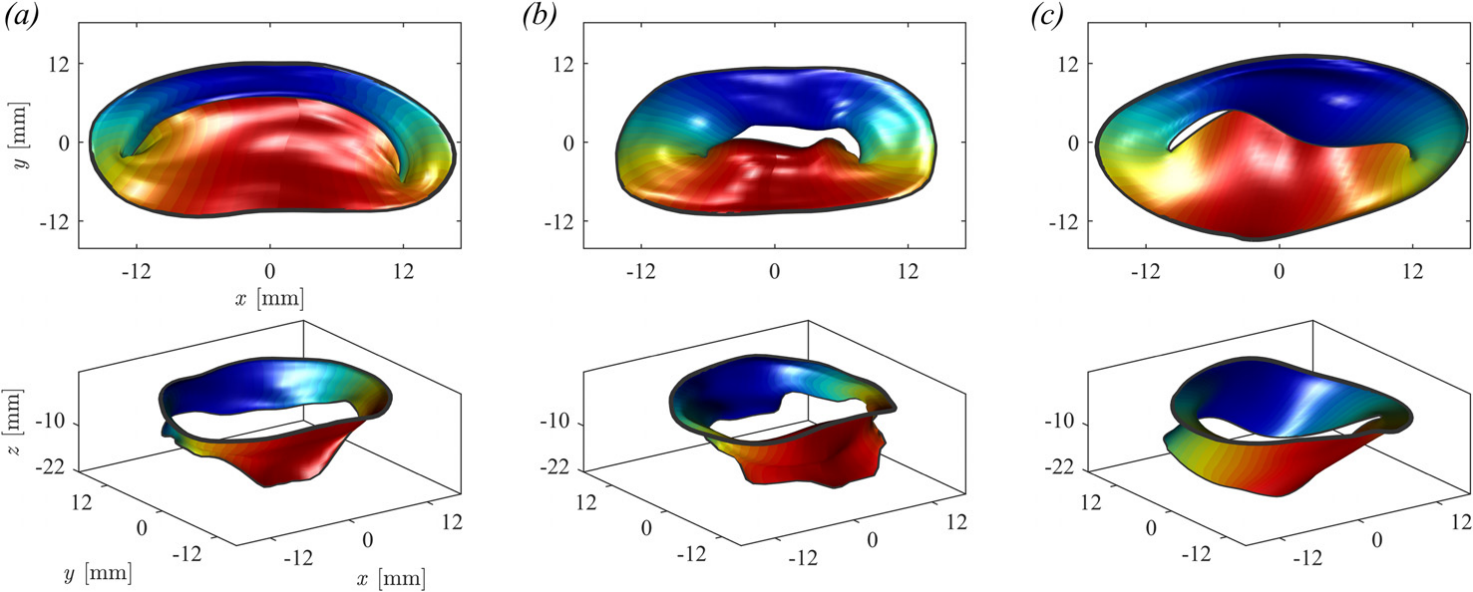
Closed and open configuration of (a) healthy MV, (b) P3 prolapse, and (c) P2 prolapse.

The MV geometries were obtained in the fully open (at peak diastole) and fully closed (during early systole) positions from clinical imaging either computed tomography (CT) or 3D-transesophageal echocardiography (TEE). Some MV geometries were obtained from CT [Figs. 6(a) and 6(b)], these were first analyzed using segmentation software (Mimics, Materialize, Leuven, Belgium) and exported to Computer-Aided Drafting (CAD)-based software for digital processing and mesh generation (3-matic, Materialize, Leuven, Belgium). Differently, geometries from TEE [Fig. 6(c)] followed a different processing procedure based on echocardiography-dedicated software (4D MV-Assessment, TomTec Imaging Systems GmbH, Unterschleissheim, Germany). In either cases, the extracted MV geometries were eventually reorganized for convenience in terms of another pair of parametric coordinates, (ϑ,s), running along the circumference and extending from the annulus to the trailing edge, respectively.

**FIG. 7.**
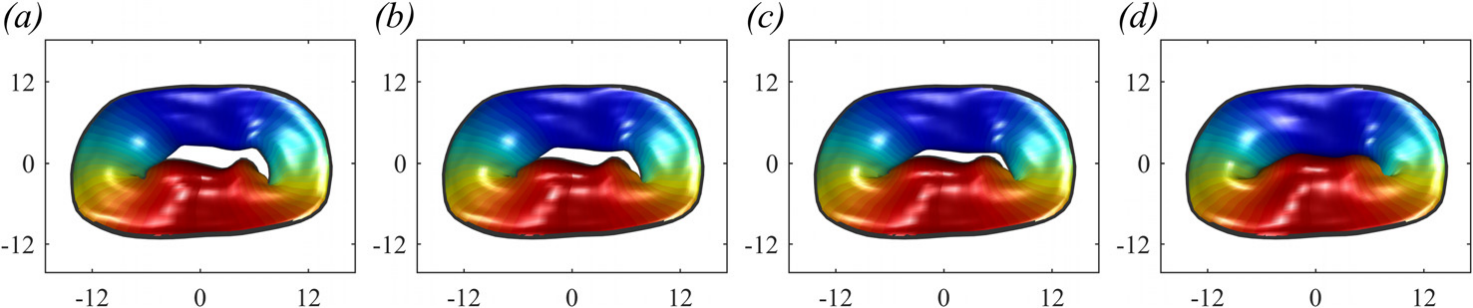
Original MV with P3 prolapse (a), modified MV with P3 prolapse reduced by 25% (b), reduced by 50% (c), and reduced by 100% (d).

**FIG. 8.**
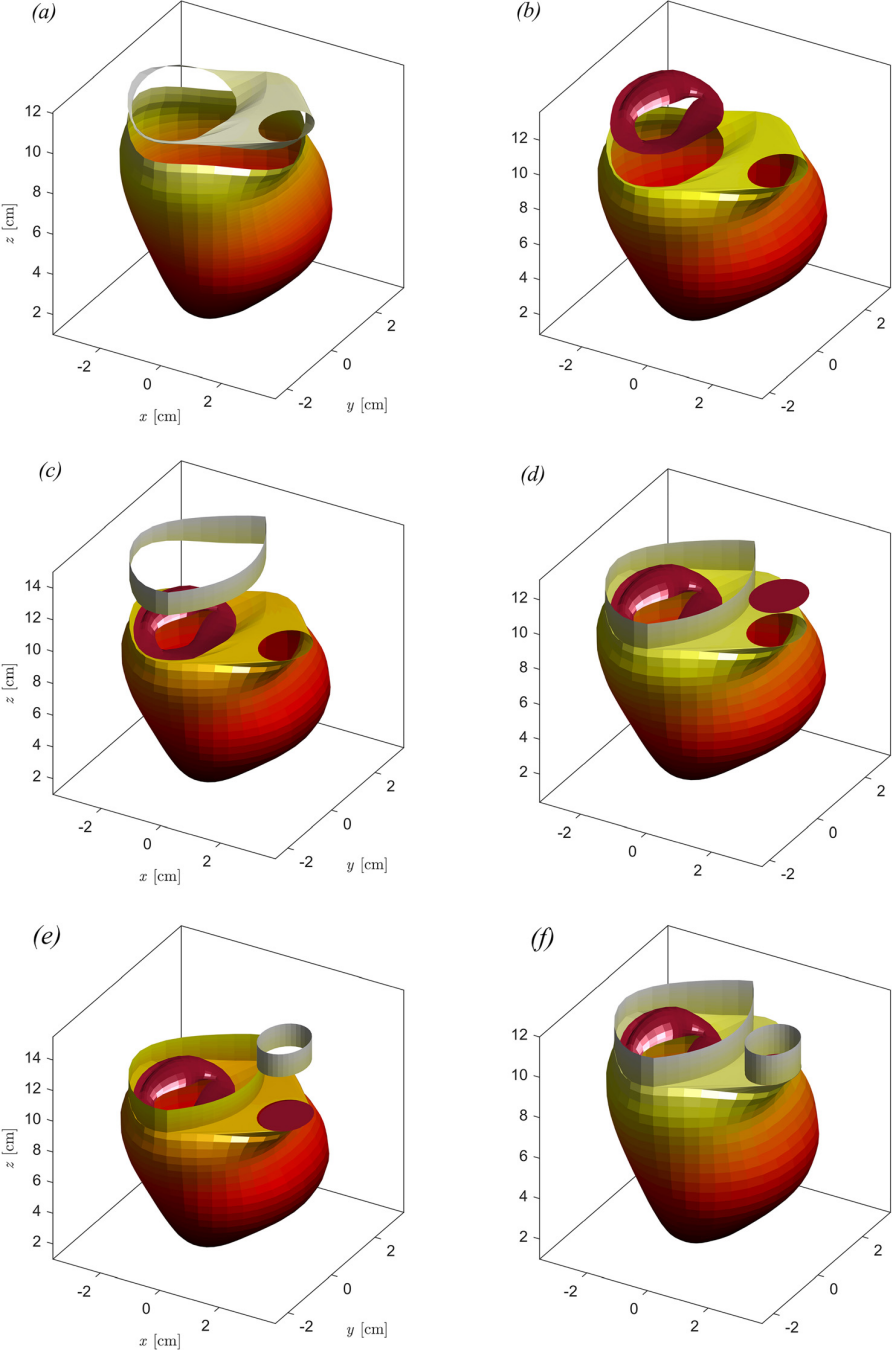
Composition of the complete geometry of dilated LV with pathological MV. (a) LV with the basal surface, (b) LV with the basal surface and pathological MV (with the leaflet semiopen), (c) LV with the basal surface, pathological MV, and atrium, (d) LV with the basal surface, pathological MV atrium, and aortic valve (closed), (e) LV with the basal surface, pathological MV atrium, aortic valve, and arteria aorta, and (f) complete geometry.

The analysis is performed on a series of MVs with different degrees and types of prolapse. On the one end, a normal geometry (called healthy-1) is used for reference, Fig. 6(a). MVP in Fig. 6(b) is a prolapse P3 of type I MR with incomplete coaptation of the leaflet and insufficient surface of coaptation of the leaflet and consequent reduction of the mitral valve area MVA. The MVP in Fig. 6(c) is a prolapse P2 of type II MR with leaflet prolapsed caused by elongation of the chordae tendineae. In addition, in order to evaluate intermediate conditions, we have artificially modified the dimension of the P3 prolapse by stretching the leaflets to let facing edges approach each other. Figure 7 shows the MV geometry at end-systole with original prolapse [P3, Fig. 7(a), equal to Fig. 7(b)], a reduction of 25% of the original prolapse [P3–25%, Fig. 7(b)], of 50% [P3–50%, Fig. 8(c)] and the 100% correction to nonregurgitating geometry [P3-healthy, Fig. 8(d)].

Once the LV and MV geometries are obtained from images, the MV and the aorta, assumed as a circular orifice, are placed at a proper position relative to the LV. These valvular elements are then connected to the LV annulus to close the valvular plane, as shown in Figs. 8(a) and 8(b). Additionally, two straight regions are added extending the aortic valve and the region around the MV (bounded by the LV annulus and the line between MV and aorta) to the upper edge of the computational domain; these represent surrogates of atrium and aorta added for numerical convenience just to avoid mixing the inflow and the outflow outside the LV, as shown in Figs. 8(c) and 8(d). An example of a complete geometry with the different composing elements is illustrated in Fig. 8. Ethical approval was not required for the anonymous geometric data used in this study.

Numerical simulations are performed with six different MVs in both healthy and pathological LVs, which allows us to compile an initial general picture of fluid dynamics properties associated with the evaluation of the regurgitation. Table V summarizes the different conditions analyzed in the present study.

**TABLE V.** List of numerical simulations with different MVs and LVs.

MV	LV	LV
Healthy	Healthy	Dilated
P3	Healthy	Dilated
P2	Healthy	Dilated
P3-25%	Healthy	Dilated
P3-50%	Healthy	Dilated
P3-Healthy	Healthy	Dilated

### B. Valve dynamic model

**FIG. 9.**
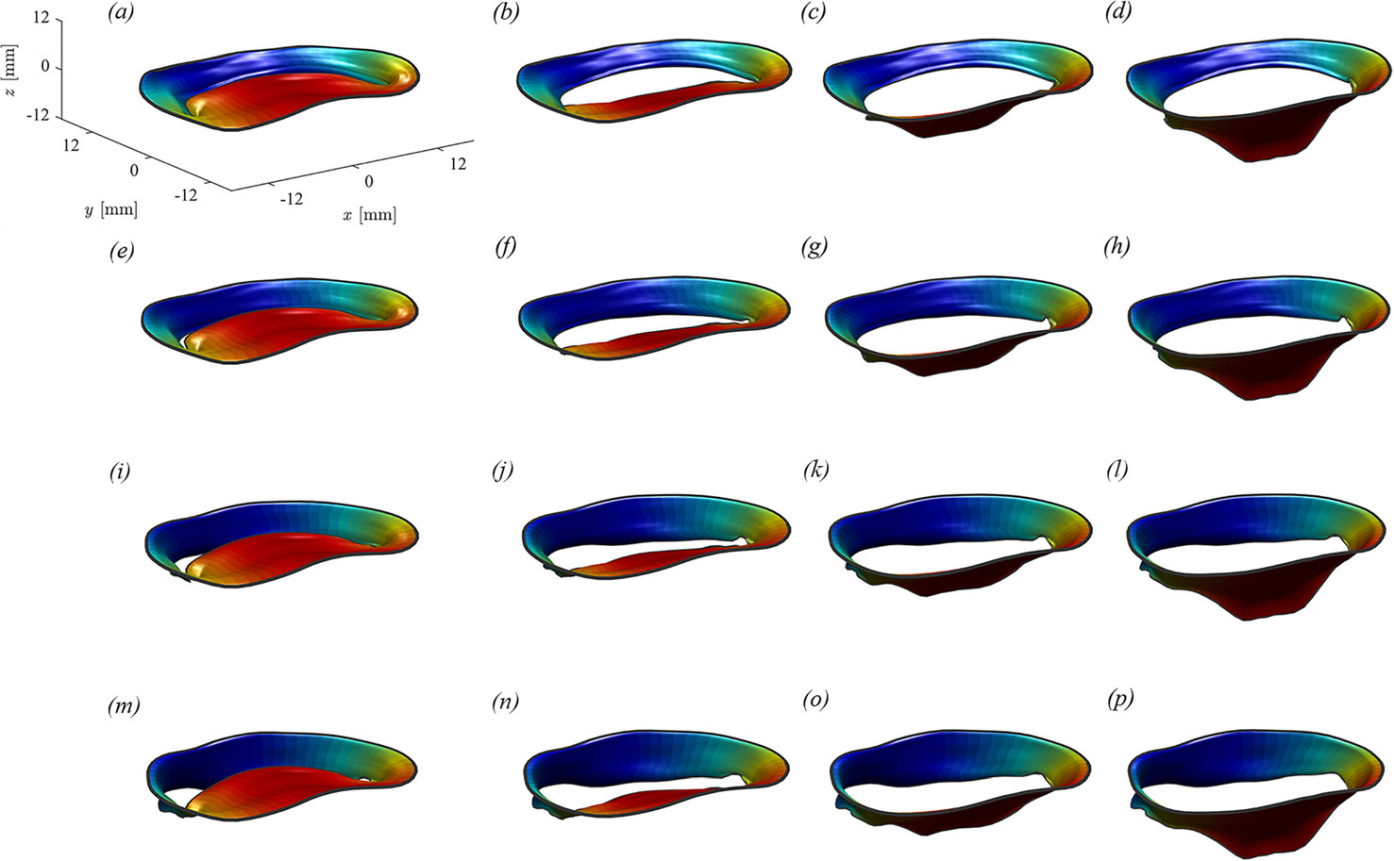
Geometry of a healthy MV for different degrees of opening of the two independent leaflets. (a) $\varphi_{1}=0,\varphi_{2}=0$; (b) $\varphi_{1}=\frac{\pi }{6},\varphi_{2}=0$; (c) $\varphi_{1}=\frac{\pi }{3},\varphi_{2}=0$; (d) $\varphi_{1}=\frac{\pi }{2},\varphi_{2}=0$; (e) $\varphi_{1}=0,\varphi_{2}=\frac{\pi }{6}$; (f) $\varphi_{1}=\frac{\pi }{6},\varphi_{2}=\frac{\pi }{6}$; (g) $\varphi_{1}=\frac{\pi }{3},\varphi_{2}=\frac{\pi }{6}$; (h) $\varphi_{1}=\frac{\pi }{2},\varphi_{2}=\frac{\pi }{6}$; (i) $\varphi_{1}=0,\varphi_{2}=\frac{\pi }{3}$; (j) $\varphi_{1}=\frac{\pi }{6},\varphi_{2}=\frac{\pi }{3}$; (k) $\varphi_{1}=\frac{\pi }{3},\varphi_{2}=\frac{\pi }{3}$; (l) $\varphi_{1}=\frac{\pi }{2},\varphi_{2}=\frac{\pi }{3}$; (m) $\varphi_{1}=0,\varphi_{2}=\frac{\pi }{2}$; (n) $\varphi_{1}=\frac{\pi }{6},\varphi_{2}=\frac{\pi }{2}$; (o) $\varphi_{1}=\frac{\pi }{3},\varphi_{2}=\frac{\pi }{2}$; and (p)$\;\varphi_{1}=\frac{\pi }{2},\varphi_{2}=\frac{\pi }{2}$.

MV imaging was best feasible in the fully open and fully closed configuration because the time resolution of current imaging technology is insufficient to reliably visualize the MV geometry during its rapid motion. Therefore, the intermediate geometric configurations were reconstructed based on general geometrical arguments as follows. The two leaflets were considered as moving independently, each one associated with a degree of opening, say $\varphi_{1}(t)$ and $\varphi_{2}(t)$, for the anterior and posterior leaflets, respectively. The valve geometry at every instant was thus described formally as $X_{v}\left(\vartheta ,s,\;\varphi_{1},\;\varphi_{2}\right)$, which represents a two-dimensional set of intermediate positions associated with the different degrees of leaflets' openings. This set of possible geometries is preliminarily estimated by interpolation between the closed $X_{v}\left(\vartheta ,s,0,0\right)$, and open $X_{v}\left(\vartheta ,s,\frac{\pi }{2},\frac{\pi }{2}\right)$ configurations obtained from images. Figure 9 shows an example of the different configurations that the healthy MV can get in correspondence with different degrees of opening of the two leaflets, $\varphi_{1}$ varying from 0 to $\frac{\pi }{2}$ from left to right and $\varphi_{2}$ increasing from top to bottom.

Once the parametric description $X_{v}\left(\vartheta ,s,\;\varphi_{1},\;\varphi_{2}\right)$ is available, a dynamic equation for the opening angles is introduced as briefly reported below; a comprehensive description and verification of the computational method, including comparison with a fluid-structure-interaction model with a given set of tissue parameters, are reported elsewhere.^4^ The valvular leaflets are assumed to move with the flow with no elastic resistance other than the constraint of remaining in the set of configurations described by the two degrees of freedom. Under this assumption, the leaflet dynamics is obtained by least-squares minimization of the difference, integrated over the valvular surface $A_{v}$, between the fluid and the valve velocity component normal to the valvular surface. This results in a 2 × 2 linear system,

$$\left[\begin{array}{cc} \iint_{A_{v}}{\left(\frac{{\partial X}_{v}}{{\partial \varphi }_{1}}\cdot n\right)}^{2}dA & \iint_{A_{v}}\left(\frac{{\partial X}_{v}}{{\partial \varphi }_{1}}\cdot n\right)\left(\frac{{\partial X}_{v}}{{\partial \varphi }_{2}}\cdot n\right)dA\\ \iint_{A_{v}}\left(\frac{{\partial X}_{v}}{{\partial \varphi }_{1}}\cdot n\right)\left(\frac{{\partial X}_{v}}{{\partial \varphi }_{2}}\cdot n\right)dA & \iint_{A_{v}}{\left(\frac{{\partial X}_{v}}{{\partial \varphi }_{2}}\cdot n\right)}^{2}dA \end{array}\right]\;\left[\begin{array}{c} \frac{{\partial \varphi }_{1}}{\partial t}\\ \frac{{\partial \varphi }_{2}}{\partial t} \end{array}\right]=\left[\begin{array}{c} \iint_{A_{v}}\left(v\cdot n\right)\left(\frac{{\partial X}_{v}}{{\partial \varphi }_{1}}\cdot n\right)dA\\ \iint_{A_{v}}\left(v\cdot n\right)\left(\frac{{\partial X}_{v}}{{\partial \varphi }_{2}}\cdot n\right)dA \end{array}\right],$$ (2)

for the two unknowns $\frac{{\partial \varphi }_{1}}{\partial t}$ and $\frac{{\partial \varphi }_{2}}{\partial t}$, where v is the fluid velocity and n is the local normal to the valvular surface.

The dynamic model described by system (2) represents an asymptotic limit of the loosest MV within the prescribed set of geometric configurations. As the model reproduces an asymptotic behavior, it does not require the introduction of mechanical parameters of the tissues that would be otherwise necessary for solving the momentum equation for the solid. This is an advantage for applications where such properties are not available; on the other hand, it should be remarked that the model results are approximate and should be read as a reference for the limiting behavior of real MVs.

This approach to valve dynamics represents a simplification with respect to a complete modeling of valvular deformation based on the actual elastic structure. Thus, this model neglects the forces due to elastic recall (although they are commonly weak in normal MVs) and reproduces the limiting behavior where the valve moves with the flow with no elastic resistance other than the constraint of moving in the prescribed set of geometrical configurations deducted from images, as shown in Fig. 9 for the normal MV. This model represents an approximation with respect to a complete calculation with fluid-structure-interaction, and it is not aimed to analyze the details of MV deformation. On the other hand, it has the advantage of not requiring a detailed definition of tissue properties that cannot be measured *in vivo*. This simplifies the solution that is aimed to reproduce the main properties of the LV fluid dynamics in the presence of a moving MV, assumed to have loose moving elements, when the general properties of the valvular structure are not available. A systematic analysis of the properties and limitations of such a valvular modeling for flow simulation is reported in a dedicated methodological study.^4^ This approach also neglects the influence of chordae tendineae whose effect is replaced by constraining the valve from opening toward the atrium.

The aortic valve, downstream the LV outlet tract, is modeled as a simple orifice with open/close behavior; the aortic valve (AV) opens when the MV is closed, and the average normal velocity at the valve position is directed toward the aorta.

### C. Numerical model

The governing equations for the flow are the Navier-Stokes equation and continuity equation

$$\frac{\partial v}{\partial t}+v\cdot \nabla v=-\nabla p+\nu \nabla^{2}v,$$ (3)

∇·v=0; (4)

where v(t,x) is the fluid velocity vector field, p(t,x) is the kinematic pressure field, and ν is the kinematic viscosity (assumed 0.04 cm^2^/s) of blood assumed as a Newtonian fluid.

The numerical solution is based on the immersed boundary method (IBM) extensively described in previous studies.^2,4–7,15,16^ Briefly, Eq. (3) is discretized in a rectangular domain using a staggered, face-centered regular Cartesian grid where spatial derivates are approximated by second-order centered finite differences. The boundary conditions at the edge of the computational box are set periodic in the x and y directions, while they are zero pressure and normal velocity on the upper and lower ends along z, respectively. The surfaces described in Sec. IV are used as immersed boundaries; to this end, the computational cells that are cut by those surfaces are assumed as impermeable and no-slip elements with a prescribed velocity vector equal to that of the corresponding immersed boundary. The time advancement of fluid velocity and valve dynamics, given by Eqs. (3) and (2), respectively, is obtained using a third-order Runge-Kutta explicit scheme. The continuity constrain (4) is then satisfied by a fractional step method by solution of a Poisson equation.^5^

### D. Flow transit analysis

The analysis of flow transit allows evaluating the properties of the washout and/or stagnation inside the LV chamber and identifying the origin of the regurgitant blood.

This analysis is performed here by solving a transport-diffusion equation for a passive scalar. Let C(x,t) be the concentration of a passive marker of particles; the diffusion-transport equation is

$$\frac{\partial C}{\partial t}+v\cdot \nabla C=\nu \nabla^{2}C,$$ (5)

which can be solved in parallel to the Navier-Stokes equation starting from the initial condition $C\left(x,0\right)=1$; in this way, the individual blood particles present in the LV at end-systole are marked. The time evolution of C allows us to verify the washout of the LV and if the blood ejected or regurgitated is made of blood that was previously present in the LV (marked by C=1) or that just arrived from the atrium (C=0).

In order to create a link with the existing literature in 4D Flow MRI, we compute the LV wash-out in terms of the residual volume,^9^ which is defined by the blood volume that was present in the LV before diastole and that is not expelled during systole. The residual volume, normalized with the end-systolic volume (ESV), can be evaluated as

$$V_{\mathrm{residual}}=\frac{1}{\mathrm{ESV}}\int_{\mathrm{ESV}}\mathrm{CdV}.$$ (6)

The increase in the residual volume corresponds to an increased possibility of blood aggregation.

### E. Regurgitation analysis

We describe here the methods used for the numerical calculation of MR properties. The effective area of the MV orifice area is computed by measuring the space between the leaflets' trailing edge

$$\mathrm{MVO}(t)=\int_{0}^{L}\left|X_{e_{\mathrm{ant}}}-X_{e_{\mathrm{post}}}\right|dL,$$ (7)

where $X_{e}=X_{v}(\vartheta ,1)$ is the trailing edge that is subdivided into the anterior and posterior leaflets and L is the length of a curve running along the midpoint between the two edges. The EOA of a regurgitant orifice is the MVO evaluated from (7) during systole when the MV is in the closed configuration.

The calculation of the blood flow rate effectively crossing the MV orifice is computed by

$$Q_{\mathrm{MV}}\left(t\right)=\int_{0}^{L}v_{\mathrm{rel}}\cdot n\left|X_{e_{\mathrm{ant}}}-X_{e_{\mathrm{post}}}\right|dL,$$ (8)

where the relative velocity, $v_{\mathrm{rel}}=\bar{v}-\frac{\partial }{\partial t}\frac{X_{e_{\mathrm{ant}}}-X_{e_{\mathrm{post}}}}{2},$ is the difference between the fluid velocity averaged along the line between the two facing edges and the velocity of the edges themselves and n is the local normal. From this, the regurgitating blood volume is the flow crossing the MVO during systole

$$V_{\mathrm{reg}}=\int_{\mathrm{sys}}Q_{\mathrm{MV}}(t)\mathrm{dt}.$$ (9)

It will also be useful, for comparison, to compute the blood volume crossing the MV annulus (MVa) to separate the contribution due to leaflet motion

$$Q_{\mathrm{MVa}}\left(t\right)=\int_{A_{\mathrm{MVa}}}\left(v_{{\mathrm{rel}}_{\mathrm{MVa}}}\cdot n\right)dA$$ (10)

where $A_{\mathrm{MVa}}$ is the area of MVa and the relative velocity $v_{{\mathrm{rel}}_{\mathrm{MVa}}}={\bar{v}}_{\mathrm{MVa}}-\frac{\partial {\bar{X}}_{\mathrm{MVa}}}{\partial t}$ is the difference between the fluid velocity averaged over the MVa and the average velocity of the annulus, with ${\bar{X}}_{\mathrm{MVa}}$ being the average position vector of the MV annulus. Then, the regurgitating blood volume measured across the MVa is

$$V_{{\mathrm{reg}}_{\mathrm{MVa}}}=\int_{\mathrm{sys}}Q_{\mathrm{MVa}}(t)\mathrm{dt}.$$ (11)

For further reference, we have also considered the flow crossing the LV annulus

$$Q_{\mathrm{LV}}\left(t\right)=\int_{A_{\mathrm{LVa}}}\left(v_{{\mathrm{rel}}_{\mathrm{LVa}}}\cdot n\right)dA,$$ (12)

where $A_{\mathrm{LVa}}$ is the area of LVa and the relative velocity $v_{{\mathrm{rel}}_{\mathrm{LVa}}}={\bar{v}}_{\mathrm{LVa}}-\frac{\partial {\bar{X}}_{\mathrm{LVa}}}{\partial t}$ is the difference between the fluid velocity averaged over the LVa and the average velocity of the annulus, with ${\bar{X}}_{\mathrm{LVa}}$ being the average position vector of the LV annulus. Then, the blood volume measured across the LVa is

$$V_{\mathrm{LVa}}=\int_{\mathrm{dia}}Q_{\mathrm{LV}}(t)\mathrm{dt}.$$ (13)

The same evaluations introduced above can be extended to define the quality of blood present in these volumes. The knowledge of the concentration field, computed by (5), allows recognizing the amount of flow that is composed of blood that was present in the LV before the beginning of diastole (hereafter indicated as *old*) or, by difference, that entered the LV during last diastole (*fresh*).

The old blood volume regurgitated by the MV orifice is obtained by extending (8) as

$$Q_{{\mathrm{MV}}_{\mathrm{old}}}\left(t\right)=\int_{0}^{L}v_{\mathrm{rel}}\cdot n\left|X_{e_{\mathrm{ant}}}-X_{e_{\mathrm{post}}}\right|C\;\mathrm{dL}\;A_{{\mathrm{MV}}_{a}},$$ (14)

where C is the concentration measured at the MV orifice; the corresponding volume is

$$V_{{\mathrm{reg}}_{\mathrm{old}}}=\int_{\mathrm{sys}}Q_{{\mathrm{MV}}_{\mathrm{old}}}\left(t\right)\;\mathrm{dt}.$$ (15)
